# Defining the impact of Getah virus envelope protein glycosylation site mutations on viral replication, host adaptation, virulence, and immune evasion

**DOI:** 10.1371/journal.ppat.1014126

**Published:** 2026-04-02

**Authors:** Tongwei Ren, Muyang Liu, Peijie Li, Liping Zhang, Zhen Zhong, Lingshan Zhou, Yifeng Qin, Kang Ouyang, Yeshi Yin, Ying Chen, Weijian Huang, Zuzhang Wei

**Affiliations:** 1 Laboratory of Animal infectious Diseases and molecular Immunology, College of Animal Science and Technology, Guangxi University, Nanning, China; 2 Guangxi Zhuang Autonomous Region Engineering Research Center of Veterinary Biologics, Nanning, China; 3 Guangxi Key Laboratory of Animal Reproduction, Breeding and Disease Control, Nanning, China; Purdue University, UNITED STATES OF AMERICA

## Abstract

The N-linked glycosylation of alphavirus envelope proteins plays critical roles in glycoprotein folding, host-receptor interactions, immune evasion, and pathogenicity. Getah virus (GETV) has two putative N-linked glycosylation sites (N-200 and N-262) in the E2 and one (N-141) in E1. We generated seven glycosylation-deficient mutants and evaluated their fitness across mammalian cells, mosquitoes, and mouse models. Loss of glycosylation at E2 N-262 or E1 N-141 enhanced in vitro replication and replication efficiency in mosquitoes, while E2 N-200 glycosylation-deficient mutants retained parental replication capacity in vitro but exhibited accelerated mosquito colonization. Despite these gains, glycan loss reduced viral adsorption/entry in selected settings and decreased measurable virion binding to MXRA8 and LDLR in vitro, while single-site E2 glycan mutants exhibited increased heparin sensitivity/affinity, indicating altered utilization of glycosaminoglycan attachment pathways. In vivo, all mutants remained lethal in 3-day-old mice but showed age-dependent attenuation in 10-day-old mice. Notably, E1 N141-deficient mutant induced no clinical symptoms and exhibited reduced viremia and tissue viral loads. Glycan ablation increased susceptibility to neutralization without impairing induction of neutralizing antibodies. Strikingly, E2 mutants rapidly reacquired glycosylation during in vivo replication, indicating strong evolutionary selection for these sites. Together, our data support an evolutionary trade-off in which GETV envelope glycans—particularly the epidemic-lineage-associated E2-N262 glycan—optimize overall fitness by balancing replication/transmission efficiency with humoral immune evasion.

## Author summary

Getah virus (GETV), an emerging mosquito-borne zoonotic alphavirus, causes significant outbreaks in animals. Epidemic strains possess a distinct N-glycosylation site (E2-N262) absent in historical lineages. The functional roles of this and conserved glycans in balancing GETV replication, transmission, virulence, and immune evasion remained unknown. This study investigates how specific glycans fine-tune GETV fitness and reveals that specific N-glycans on GETV envelope proteins critically balance viral traits. Ablating E2-N262 or E1-N141 enhanced replication in vitro and in mosquitoes, while E2-N200 loss accelerated early mosquito colonization. However, all mutants showed severely attenuated virulence in 10-day-old mice. Crucially, removing E2 glycans, especially at N262, increased susceptibility to neutralizing antibodies, confirming glycan shielding. A key discovery was the rapid in vivo reversion of E2 glycosylation-deficient mutants back to the glycosylated state in mice, driven by strong selective pressure (highest for epidemic-lineage E2-N262). Thus, these glycans fine-tune GETV replication, transmission, pathogenesis, and immune evasion, with evolutionary pressure favoring their retention to optimize the replicative efficiency-immune escape trade-off in epidemic strains.

## 1.  Introduction

Getah virus (GETV), a mosquito-borne alphavirus within the Togaviridae family, poses an emerging threat to animal and potential human health across Eurasia and Southeast Asia [1]. First isolated in 1955 from *Culex gelidus* mosquitoes in Malaysia, GETV has since demonstrated a remarkable capacity to expand its geographic distribution and host range [2,3]. While pigs and horses serve as primary amplification hosts, linked to outbreaks of fever, reproductive failure, and encephalitis [4,5], infections have also been documented in diverse vertebrates, including cattle, foxes, wild boars, sheep, primates, and avian species [6–11]. Notably, serological evidence suggests zoonotic potential: neutralizing antibodies against GETV have been detected in human sera, with significantly higher titers observed in febrile patients compared to healthy humans [12,13].

The GETV genome comprises a single-stranded, positive-sense RNA of ~11.7 kb, flanked by 5′- and 3′-untranslated regions (UTRs) and a poly-A tail. Two open reading frames (ORFs) encode nonstructural (nsP1-nsP4) and structural proteins (Capsid, E3, E2, 6K, and E1) [14,15]. The nsPs orchestrate viral replication and host immune modulation, while the structural proteins assemble into heterotrimeric E2-E1 spikes that mediate receptor binding, membrane fusion, and virion budding [16–18]. E2, the immunodominant glycoprotein, drives neutralizing antibody responses and interacts directly with the nucleocapsid core, positioning it as a critical determinant of infectivity and immunogenicity [19].

Glycosylation, a ubiquitous post-translational modification, serves critical functions in diverse biological processes [20]. N-linked glycosylation specifically targets asparagine residues within the conserved Asn-X-Ser/Thr motif (where X denotes any amino acid except proline) [21]. Viral proteins, particularly envelope glycoproteins, have been extensively studied in this context, as their N-linked glycosylation is indispensable for proper folding, intracellular trafficking, membrane fusion, and receptor-binding activity [22]. Notably, in pathogens such as human immunodeficiency virus (HIV), severe acute respiratory syndrome coronavirus (SARS-CoV), hepatitis C virus (HCV), and simian immunodeficiency virus (SIV), N-linked glycans on envelope proteins modulate neutralization sensitivity by shielding antigenic epitopes, thereby facilitating immune evasion [23–26].

Among alphaviruses, envelope glycoproteins exhibit conserved N-linked glycosylation sites, though their quantity and spatial distribution vary significantly across viral species [27–30]. Prior studies have established that N-linked glycosylation in alphaviruses regulates glycoprotein biosynthesis, viral replication efficiency, host-cell interactions, and immune recognition [31–33]. Recent advances further demonstrate that N-glycans mediate binding to the MXRA8 receptor through mechanisms dependent on structural topology, directly impacting viral tropism and oncolytic efficacy [34]. These findings underscore the dual role of N-linked glycosylation in balancing viral fitness and immune evasion, highlighting its therapeutic relevance in antiviral strategies and oncotherapy. Despite these advances, the functional significance of N-glycosylation in GETV glycoproteins remains unexplored—a critical gap, given the expanding burden of GETV-associated disease.

GETV phylogeny delineates four distinct evolutionary lineages (Groups I-IV), with Groups I (GI) and II (GII) comprising historical isolates, while Group III (GIII) represents the predominant epidemic lineage encompassing nearly all global isolates since the 1960s. Notably, GIII exhibits broader vector diversity, expanded host tropism, and exclusive association with epizootic outbreaks in animals, distinguishing it from other lineages [35]. Despite its epidemiological dominance, the genetic determinants underlying variations in GETV virulence and transmission efficiency, as well as their mechanistic roles in disease progression, immune evasion, and epidemic spread, remain poorly characterized. Comparative genomic analyses reveal that GIII, along with GII and GIV, uniquely acquired a novel N-linked glycosylation site at position 262 of the E2 glycoprotein (E2-N262)—a feature absent in the prototype GI strains [36]. This lineage-specific gain raises critical questions: Does E2-N262 glycosylation contribute to the heightened pathogenicity and epidemic fitness of GIII viruses? Furthermore, the biological roles of the conserved glycosylation sites at E1-N141 and E2-N200 across all GETV lineages remain uncharacterized, warranting systematic investigation into their functional significance in viral adaptation and pathogenesis.

Here, we systematically ablated glycosylation sites in the E1 and E2 glycoproteins and generated a panel of recombinant GETV variants to dissect the contributions of specific glycans to viral replication, host adaptation, virulence and neutralization resistance. Our findings reveal that site-specific N-glycosylation in GETV E1 and E2 glycoproteins critically balances viral replication efficiency, mosquito colonization, mammalian virulence, and immune evasion by modulating sensitivity to neutralizing antibodies. These results provide mechanistic insights into GETV’s evolutionary trajectory and inform the rational design of vaccines and therapeutics targeting this neglected arbovirus.

## 2.  Materials and methods

### 2.1.  Ethics statement

The animal experiments were approved by the Animal Experiment Committee of Guangxi University with the approval number (GXU2022-288).

### 2.2.  Cells, plasmids, and antibodies

BHK-21 cells (ATCC, CCL-10, RRID: CVCL_1914) and Vero cells (ATCC, CCL-81, RRID: CVCL_0059) were purchased from American Type Culture Collection (ATCC) and cultured using established protocols. C6/36 cells (ATCC, CRL-1660, RRID: CVCL_Z230) were purchased from ATCC and maintained in Dulbecco’s Modified Eagle Medium (DMEM, Gibco) supplemented with 10% fetal bovine serum (FBS, Gibco) and L-glutamine at 28°C under 5% CO₂. BHK-21 cells, which are highly permissive to alphavirus infection and transfection, were used for plasmid transfection, virus rescue, and initial propagation. Vero cells, which form uniform monolayers and are defective in interferon signaling, were used for plaque assays and studies requiring a standardized system for quantifying infectious virus and cytopathic effects. C6/36 cells, derived from *Aedes albopictus* mosquitoes, were used to model viral replication in the invertebrate host. The full-length infectious cDNA clone (pGETV-GX) of GETV strain GX201808 (GenBank accession no. MT269657), along with monoclonal antibodies (mAbs) against GETV E1 and GETV Cap, were generated as previously described [37–39]. The GETV-E2 mAb was maintained in our laboratory.

### 2.3.  Plasmid constructs

N-glycosylation sites within the E1 and E2 glycoproteins were predicted using the NetNGlyc 1.0 server. Three potential sites were identified: one in E1 (¹⁴¹NQT¹⁴³) and two in E2 (²⁰⁰NCT²⁰² and ²⁶²NST²⁶⁴). Seven mutant GETV cDNA clones were engineered by substituting asparagine (N) with glutamine (Q) within the Asn-X-Ser/Thr motifs at E2-200, E2-262, and/or E1-141 to disrupt N-glycosylation. For the E2-N200 glycosylation mutant, SrfI and BstBI restriction sites in pGETV-GX were selected to amplify the target region. Primers SrfI-F and BstbI-R were designed to generate overlapping fragments via splice overlap extension PCR (SOE-PCR). Point mutations were introduced using primers E2-200F/E2-200R, amplifying two fragments (SrfI-F/E2-200R and E2-200F/BstbI-R). These fragments were fused by SOE-PCR, digested with SrfI and BstBI, and ligated into the corresponding sites of pGETV-GX to generate rE2-200Q. Single-site mutants (rE1-141Q and rE2-262Q) were constructed analogously using primers listed in S1 Table. Double and triple sites mutants were subsequently generated using single-site mutants as templates.

### 2.4.  DNA transfection and virus passage

BHK-21 cells were seeded into six-well plates and cultured at 37°C under 5% CO₂. Upon reaching 80% confluence, cells were transfected with 2 μg of plasmid DNA using Lipofectamine 2000 (Thermo Fisher Scientific, Waltham, MA, USA) according to the manufacturer’s protocol. Cell culture supernatants were collected at 48 hours post-transfection (hpt) and designated as passage 0 (P0) virus stock. P0 viruses were inoculated into fresh BHK-21 and C6/36 cells for serial propagation. Cytopathic effects (CPE) were monitored daily; when approximately 90% of the cell monolayer exhibited CPE, supernatants were harvested and labeled as passage 1 (P1). P1 viruses were diluted 1:1000 in culture medium and used to infect fresh cells. Subsequent passages (P2-P3) were performed using identical procedures. Viral RNA from P3 stocks was extracted, and the E protein-coding regions were sequenced via Sanger sequencing. After centrifugation, the viral particles were lysed and divided into two aliquots. One aliquot was treated with Peptide N-Glycosidase F (PNGase F) (New England BioLabs, Inc.) according to the manufacturer’s instructions. Finally, both treated and untreated viral proteins were analyzed by Western blot to compare their molecular weights.

### 2.5.  Immunofluorescence assay (IFA)

BHK-21 cells were seeded in six-well plates and grown to 90% confluence. Cells were infected with specified viruses at a multiplicity of infection (MOI) of 0.1, while uninfected cells served as negative controls. At 18 hpi, supernatants were removed, and cells were washed twice with phosphate-buffered saline (PBS), followed by fixation in ice-cold 4% formaldehyde for 15 minutes at −20°C. Fixed cells were permeabilized with 0.1% Triton X-100, blocked with 5% bovine serum albumin (BSA) in PBS for 30 minutes at 37°C, and incubated with anti-GETV-E2 monoclonal antibody (1:1000 dilution) for 2 hours at 37°C. After three washes with PBS, cells were incubated with Alexa Fluor 568-conjugated goat anti-mouse IgG (H&L) as secondary antibody (1:500 dilution) for 1 hour at 37°C. Nuclei were counterstained with 4′,6-diamidino-2-phenylindole (DAPI) for 15 minutes. Fluorescence images were acquired using an inverted fluorescence microscope (Nikon Eclipse Ti, Shanghai, China).

### 2.6.  Western blotting (WB)

BHK-21 cells cultured in six-well plates were infected with specified viruses (MOI = 1). At 24 hpi, cells were lysed using RIPA buffer (CW Biotech, Beijing, China) supplemented with protease inhibitors. Lysates were centrifuged at 15,000×g for 10 minutes at 4°C to pellet debris. Supernatants were mixed with 5× SDS-PAGE loading buffer, denatured at 95°C for 10 minutes, and resolved on 10% SDS-polyacrylamide gels. Proteins were electrophoretically transferred onto polyvinylidene fluoride (PVDF) membranes (Millipore, Burlington, MA, USA). Membranes were blocked with 5% non-fat milk in Tris-buffered saline containing 0.1% Tween-20 (TBST) for 2 hours at 37°C, then probed separately with GETV-E2, GETV-E1 and GETV-Cap monoclonal antibody (1:1000 dilution) for 2 hours at 37°C. After three TBST washes, membranes were incubated with horseradish peroxidase (HRP)-conjugated goat anti-mouse IgG (1:5000 dilution) for 1 hour at 37°C. Protein bands were visualized using an enhanced chemiluminescence (ECL) detection system (Bio-Rad, Hercules, CA, USA).

### 2.7.  Plaque assay

Vero cells were seeded into six-well plates and grown to 100% confluence. Viral samples were serially diluted (10-fold) in 2% DMEM. For each dilution, 100 μL of inoculum was applied to triplicate wells and incubated at 37°C for 1 hour. Cells were washed with PBS to remove unbound virus, then overlaid with DMEM containing 1% (w/v) low-melting-point agarose (Cambrex, Rockland, ME, USA) and 1% penicillin/streptomycin. Plates were incubated at 37°C for 60 hours, after which cells were fixed with 3.7% formaldehyde in PBS for 6 hours at room temperature. Following fixation, agarose plugs were carefully removed, and monolayers were stained with 1% crystal violet in 20% ethanol for 15 minutes. Plaques were enumerated visually, and viral titers were expressed as plaque-forming units per milliliter (PFU/mL).

### 2.8.  TCID_50_ titration and viral growth kinetics

For TCID_50_ determination, viruses were serially diluted (10-fold) in 2% DMEM and inoculated onto confluent Vero cells in 96-well plates. After 1 hour of adsorption at 37°C under 5% CO₂, inocula were replaced with fresh 2% DMEM. CPE were monitored daily for 5 days, and TCID_50_ values were calculated using the Reed-Muench method.

For growth kinetics, a low MOI of 0.01 was used to allow for multiple rounds of replication, thereby amplifying differences in replication efficiency between viruses. In contrast, a high MOI of 5 was used for single-cycle infection assays to synchronize the infection. BHK-21, Vero and C6/36 cells in 12-well plates were infected with indicated viruses at a multiplicity of infection (MOI) of 0.01 (triplicate wells per time point). After 1 hour of adsorption at 37°C (BHK-21 and Vero) or 28°C (C6/36), cells were washed twice with PBS and replenished with 2% DMEM. Supernatants were collected at 12, 18, 24, 36, and 48 hpi, and viral titers were quantified via TCID_50_ assay. Multistep growth curves were generated by plotting log10 TCID_50_/mL against time.

### 2.9.  Animal experiments

All procedures were approved by the Animal Ethics Committee of Guangxi University (Protocol No. GXU2022-288). Specific pathogen-free (SPF) 3- and 10-day-old ICR mice were randomly divided into experimental and control groups. Mice were subcutaneously inoculated with 10⁴ TCID_50_ of WT virus or mutant viruses in 50 μL DMEM; control groups received sterile DMEM.

For morbidity assessment (n = 10 per group), mice were monitored daily for 14 days for clinical symptoms, including weight loss, lethargy, respiratory distress, limb paralysis, or mortality. Clinical scores were assigned as follows: 0 (asymptomatic), 1 (lethargy/respiratory distress), 2 (limb paralysis/diarrhea), or 3 (moribund state or euthanasia). Moribund mice were humanely euthanized via CO₂ asphyxiation.

For histopathological evaluation, brain tissues and knee joint samples were fixed in 4% paraformaldehyde. Prior to embedding, knee joints underwent decalcification using EDTA. Tissues were sequentially dehydrated through graded ethanol, cleared in xylene, and embedded in paraffin blocks. Serial sections of 4–6 μm thickness were cut, mounted on microscope slides, and stained with hematoxylin and eosin (H&E) for microscopic analysis.

For viral load analysis (n = 15 per group), mice were euthanized at 1, 3, and 5 days post-infection (dpi; n = 5 per time point). Blood, lung, knee joint, and brain tissues were harvested, weighed, and homogenized in PBS (10% w/v) for subsequent viral titration.

### 2.10.  Viral load quantification

Tissue homogenates were subjected to three freeze-thaw cycles, followed by centrifugation at 15,000×g for 10 minutes at 4°C. Clarified supernatants were serially diluted in 2% DMEM, and viral titers in tissues were determined via TCID_50_ assay as described in Section 2.7. Results were normalized to tissue weight and expressed as log10 TCID_50_ per 0.1 gram (log10 TCID_50_/0.1g).

### 2.11.  Virus attachment assay

Confluent cell monolayers in 24-well plates were pre-chilled at 4°C for 10 minutes and inoculated with pre-cooled virus (MOI = 5) to facilitate binding. A high MOI of 5 was used for attachment and entry assays to ensure a strong signal-to-noise ratio for bound virions. Cells were incubated at 4°C for 1 hour, washed three times with cold PBS, and lysed with 500 μL TRIzol reagent (Invitrogen) for RNA extraction. Viral attachment was quantified via quantitative reverse transcription PCR (RT-qPCR) targeting the E2 gene. Viral genome copy numbers were determined using a standard curve and normalized to the rGETV-GX control group.

### 2.12.  Virus entry assay

Confluent cell monolayers in 24-well plates were inoculated with mutant or WT viruses (MOI = 5) and incubated at 4°C for 1 hour. Cells were washed with a cold alkaline high-salt solution (1 M NaCl and 50 mM sodium bicarbonate, pH 9.5) for 3 minutes to remove non-internalized virus. After adding DMEM, cells were incubated at 37°C/28°C for 30 minutes. A second wash with the cold alkaline high-salt solution was performed to remove non-internalized viruses. Viral entry efficiency was assessed by RT-qPCR quantification of the E2 gene in lysed cells.

### 2.13.  Heparin inhibition assay

Heparin (Hp; 2 mg/mL, Solarbio) sensitivity was evaluated as previously described [40]. Viruses were pre-incubated with an equal volume of Hp or PBS (control) at 37°C for 1 hour, followed by chilling on ice for 10 minutes. Pre-cooled Vero cell monolayers (4°C, 10 minutes) were inoculated with treated or untreated virus, adsorbed at 4°C for 1 hour, and washed to remove unbound particles. RNA was isolated using TRIzol, and adsorbed viral genomes were quantified by RT-qPCR, normalized to untreated controls.

For plaque-based binding assessment, Hp-treated viruses (MOI = 0.01) were adsorbed to pre-chilled cells (4°C, 1 hour). Plaque assays were performed as in Section 2.6, with plaque counts reflecting heparin-bound infectious particles.

### 2.14.  Neutralization assay

Neutralization sensitivity was tested using twofold serial dilutions of heat-inactivated serum mixed with 200 TCID_50_ of virus. After incubation (37°C, 2 hour), mixtures were adsorbed onto BHK-21 cells for 1 hour, replaced with 2% DMEM, and CPE were monitored at 3 days post-infection (dpi). The 50% neutralizing dose (ND_50_) was calculated using the Reed-Muench method [41].

### 2.15.  Mosquito infection studies

*Aedes albopictus* mosquitoes were reared at 28°C and 80% relative humidity, fed 10% sucrose, and starved for 24 hours pre-infection. Virus-defibrinated sheep blood-sucrose mixtures (1:1:1 ratio) were applied to sponge feeders. Five- to seven-day-old mosquitoes were allowed to feed in darkness. Engorged females were isolated and maintained under controlled conditions. Viral loads in mosquitoes were quantified via RT-qPCR at 5 and 10 dpi.

### 2.16.  Statistical analysis

Data are expressed as mean ± standard deviation (SD). Unpaired Student’s *t*-tests (GraphPad Prism 8.0.2) determined significance between groups, with thresholds set as *p < 0.05 (*), **p < 0.01 (**), ***p < 0.001 (***), and ****p < 0.0001 (****).

### 2.17.  Molecular modeling of the GETV E glycoprotein

Based on the provided sequences and using the GETV Cryo-EM structure (PDB: 7WC2) as a reference, three-dimensional structural modeling was performed via the SWISS-MODEL server (https://swissmodel.expasy.org). Subsequently, the constructed E1-E2 model was used as a ligand and docked with the predicted structure of the receptor protein MXRA8 (UniProtKB: Q9DBV4) and LDLR (UniProtKB: P35951, obtained from AlphaFoldDB) using the GRAMM Web server (https://gramm.compbio.ku.edu). The docking results were visualized and the interaction interfaces were analyzed using PyMOL (v3.1.6.1, Schrödinger, LLC) and PDBePISA (https://www.ebi.ac.uk/pdbe/pisa/).

### 2.18.  Surface plasmon resonance (SPR) experiments

Surface plasmon resonance (SPR) experiments were performed using a Biacore 8K system (Cytiva) equipped with a CM5 sensor chip. The chip surface was activated with EDC/NHS (400 mM EDC and 100 mM NHS mixed 1:1) at 10 μL/min for 420 s. Mouse MXRA8 or LDLR (20 μg/mL) was immobilized on the sample flow cell (Fc2) at 10 μL/min to a level of approximately 12,600 RU, while the reference flow cell (Fc1) was left unmodified. Remaining active esters were blocked with 1 M ethanolamine (pH 8.5) at 10 μL/min for 420 s. For kinetic analysis, purified virions were serially diluted and injected sequentially in PBS-T running buffer at 20 μL/min, with an association phase of 100 s and a dissociation phase of 180 s. After each cycle, the sensor surface was regenerated. Binding data were analyzed using Biacore Insight software and fitted to a 1:1 Langmuir binding model.

## 3.  Results

### 3.1.  Identification of N-linked glycosylation sites in getv envelope proteins and generation of glycosylation-deficient mutants

To define the functional landscape of N-glycosylation in GETV, we first sought to identify and genetically ablate all predicted glycosylation sites on its envelope proteins.

**Fig 1:**
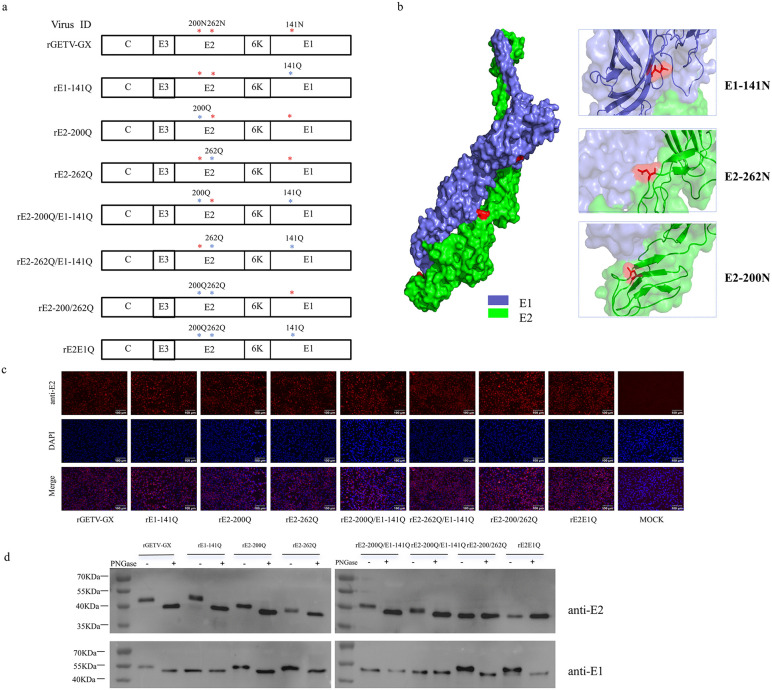
Construction and identification of glycosylation site mutant virus. **(a)** Schematic illustration of N-glycosylation mutant generation. Asterisks denote predicted N-linked glycosylation sites in E1 and E2. Mutants were constructed via SOE-PCR site-directed mutagenesis. Mutated residues are indicated (*). Fragments were sequence-verified and cloned into the pGETV-GX plasmid backbone. **(b)** The E1-E2 dimer structure is displayed based on SWISS-MODEL server using cryo-EM structure of GETV (PDB: 7WC2) as the template. Protein domains are color-coded: E1 is shown in sky blue; E2 is shown in green. E2-N200, E2-N262 and E1-N141 are displayed in red. **(c)** IFA confirming E2 protein expression in rescued mutants. Cells infected with WT virus or mutant viruses (MOI = 0.1) were fixed at 18 hpi and probed with GETV E2-specific monoclonal antibody (mAb). **(d)** Endoglycosidase analysis of E protein. The collected viral supernatant was centrifuged and lysed, then digested with PNGase F according to the manufacturer’s instructions, followed by Western blot analysis using E2 mAb and E1 mAb. The size differences of E protein reflect its glycosylation levels.

Bioinformatic analysis (NetNGlyc 1.0) of the GETV GX201808 strain revealed two putative N-linked glycosylation sites in the E2 glycoprotein (²⁰⁰NCT²⁰² and ²⁶²NST²⁶⁴) and one in E1 (¹⁴¹NQT¹⁴³), the locations of which are consistent with surface-exposed residues identified in published GETV cryo-EM structures (Fig 1a). To investigate the functional role of these sites, asparagine (N) residues within the Asn-X-Ser/Thr motifs were substituted with glutamine (Q) via codon mutagenesis (AAT/AAC → CAA). These substitutions abrogate glycosylation without altering the physicochemical properties of the sequons. Mutants were designated based on the modified residue (e.g., rE2-200Q, rE2-262Q, rE1-141Q) and validated by Sanger sequencing. A schematic of mutant constructs is shown in Fig 1a. Using the existing GETV cryo-EM structure, we simulated the E1-E2 dimer with SWISS-MODEL server, which predicted that all three glycans are surface-exposed: N141 at the tip of a β-hairpin, and N200 and N262 at the turn (Fig 1b).

### 3.2. Mutations in N-linked glycosylation sites do not affect the recovery of infectious viruses in BHK-21 cells

Before assessing functional phenotypes, it was crucial to determine whether the ablation of glycosylation sites impacts the fundamental processes of viral assembly and polyprotein processing, which could confound subsequent interpretations. Transfection of recombinant plasmids into BHK-21 cells yielded infectious progeny viruses for all mutants. Pronounced CPE were observed in BHK-21 cells infected with P0 supernatants at 24 hpi (Fig 1b). Serial passaging (P1-P3) confirmed stable propagation of mutants in BHK-21 cells. Immunofluorescence assays (IFA) using anti-E2 mAb demonstrated robust E2 expression in cells infected with P3 viruses, confirming successful rescue and antigenic integrity of mutant viruses (Fig 1c). Sequencing of P3 viral RNA confirmed retention of engineered mutations (Fig 1c).

Western blot analysis revealed altered electrophoretic mobility of E1 and E2 glycoproteins in mutants compared to WT virus, consistent with loss of glycosylation (Fig 1d). For example, the rE2-200Q mutant exhibited faster migration, confirming ablation of the N200 glycosylation site. Similar shifts were observed for other mutants. We performed PNGase F treatment to further confirm that the observed mobility shifts of the E2 and E1 proteins were indeed due to glycan modifications rather than protein truncation (Fig 1d). Western blot analysis combined with PNGase F treatment confirmed that all three predicted sites (E1-141, E2-200, and E2-262) are authentic N-glycosylation motifs. The successful rescue of all mutants confirmed that none of these glycosylation sites are essential for viral viability, allowing us to proceed with a systematic functional dissection.

### 3.3. Replication kinetics of glycosylation-deficient mutants in mammalian and mosquito cells

**Fig 2:**
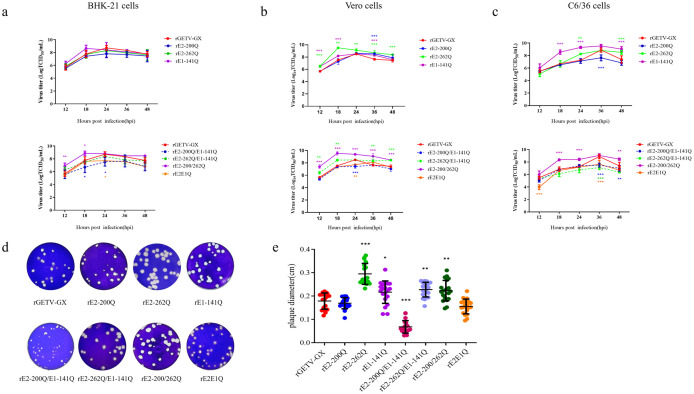
In vitro replication kinetics of N-glycosylation-deficient viruses in mammalian and mosquito cell lines. **(a-c)** Multi-step growth curves of WT virus and mutant viruses in BHK-21 cells, Vero cells, and C6/36 cells. Cells were infected with indicated viruses at a multiplicity of infection (MOI) of 0.01 (triplicate wells per time point). After 1 hour of adsorption at 37°C (BHK-21 and Vero) or 28°C (C6/36), cells were washed twice with PBS and replenished with 2% DMEM. Supernatants were harvested at 12, 18, 24, 36, and 48 hpi, with titers determined by TCID_50_/mL assay. **(d-e)** Plaque morphology and diameter analysis in Vero cells. Error bars denote standard deviation (SD) from three biological replicates. Statistical significance: *p < 0.05; **p < 0.01; ***p < 0.001 (Student’s *t*-test).

We next sought to determine how site-specific glycans influence GETV fitness across different host environments. We hypothesized that these glycans might differentially regulate replication in vertebrate (BHK-21, Vero) and invertebrate (C6/36) cells, reflecting adaptations required for its arboviral life cycle. In BHK-21 cells, single glycosylation-deficient mutants (rE2-200Q, rE2-262Q, rE1-141Q) replicated with kinetics comparable to WT virus. rE1-141Q showed a transient increase in early replication (12-18 hpi), though not statistically significant. The double E2 mutant (rE2-200/262Q) exhibited accelerated early replication (12-18 hpi; *p* < 0.05), while rE2-200Q/E1-141Q displayed reduced titers from 18-24 hpi. The triple glycosylation sites mutant (rE2E1Q) peaked earlier (18 hpi) but at lower titers than WT virus (Fig 2a).

In Vero cells, rE1-141Q and rE2-262Q replicated more efficiently than WT virus (*p* < 0.01), while rE2-200Q showed no significant difference. rE2-200/262Q achieved titers exceeding WT virus at all timepoints (*p* < 0.001). Conversely, rE2E1Q and rE2-200Q/E1-141Q showed attenuated peak titers (Fig 2b).

In C6/36 cells, rE1-141Q and rE2-200/262Q replicated efficiently, maintaining higher titers than WT virus. rE2E1Q and rE2-200Q/E1-141Q exhibited delayed replication (12 hpi) and reduced peak titers (36 hpi). rE2-262Q matched WT virus replication early but surpassed WT virus by 24 hpi (*p* < 0.05), retaining higher titers at 48 hpi. rE2-262Q/E1-141Q showed significantly impaired replication (Fig 2c).

Plaque phenotypes for mutants were determined in Vero cells. All mutants formed distinct plaques in Vero cells. rE2E1Q and rE2-200Q produced plaques comparable in size to WT virus. However, rE2-200Q/E1-141Q generated smaller plaques (*p* < 0.01), while other mutants (rE2-262Q, rE1-141Q, and rE2-200/262Q) formed larger plaques (*p* < 0.05; Fig 2d-e). These results demonstrate that specific glycan losses (e.g., at E2-N262 and E1-N141) can potentiate GETV replication in a cell-type-dependent manner, recasting glycosylation not as a binary requirement but as a nuanced regulator of viral replication.

### 3.4. The removal of E-glycosylation perturbs early infection steps and virion infectivity

To investigate the mechanistic basis for the increased infectious virus yield from E-glycosylation mutants in Vero cells, we systematically evaluated the impact of these mutations on both the early (attachment and entry) and late (virion assembly and release) stages of the viral life cycle.

**Fig 3:**
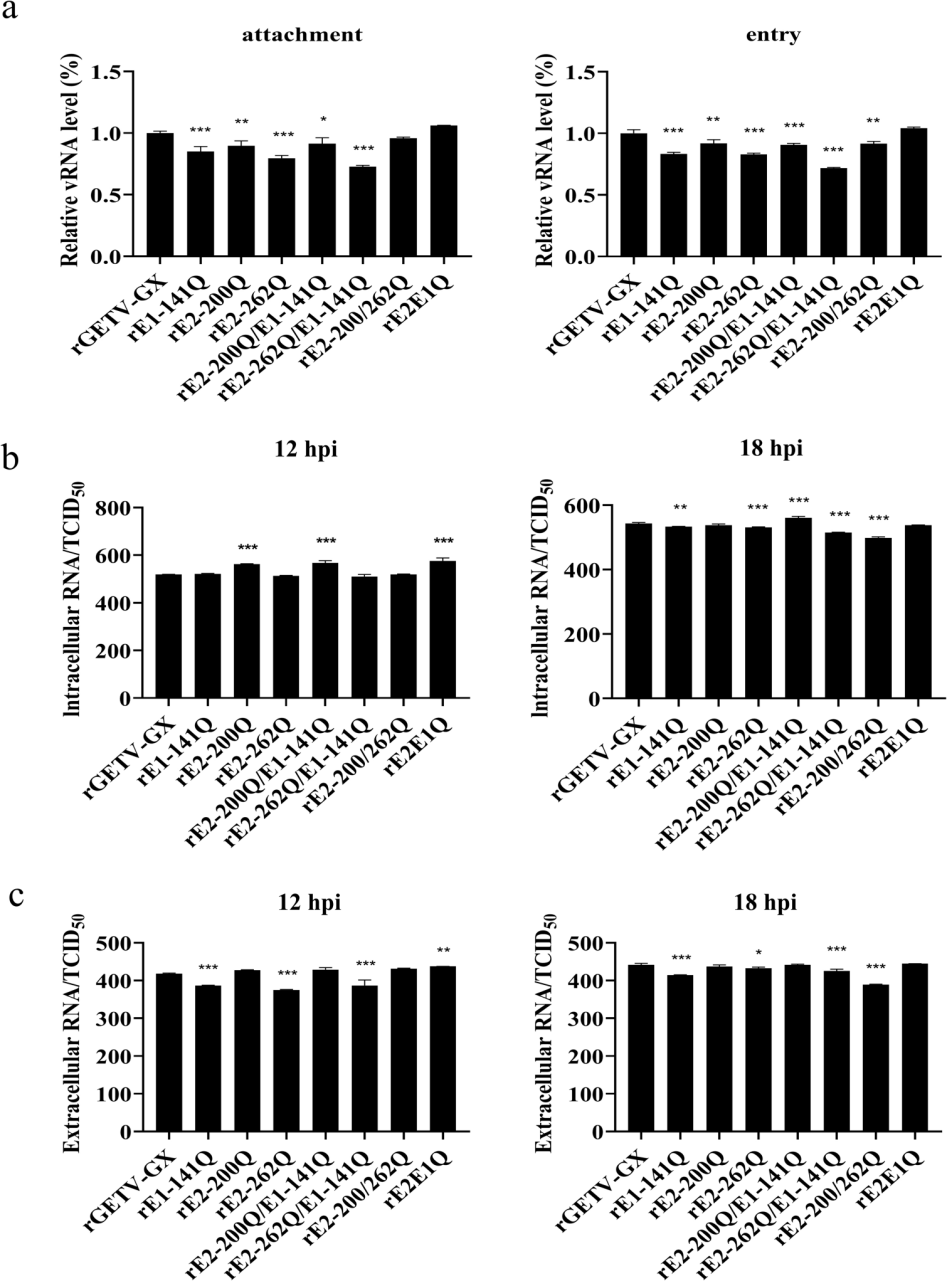
Effects of N-glycosylation mutation on GETV life cycle in Vero cells. **(a)** Attachment (4°C, 1 hour) and entry (4°C for 1 hour and then 37°C/28°C for 30 minutes) efficiencies of WT virus and mutant viruses (MOI = 5). Viral genome copies were quantified by RT-qPCR. **(b-c)** Intracellular and extracellular RNA/TCID_50_ ratio. Vero cells were infected with WT or mutant virus at an MOI of 5. After infection, the viral inoculum was removed, and the cells were washed three times with PBS. To quantify intracellular viral RNA at 12 hpi and 18 hpi, the cells were further rigorously washed with an alkaline high-salt solution before sample collection. Intracellular and extracellular viral RNA were measured by RT-qPCR, and infectious virus was determined by TCID_50_ assay.

In BHK-21 cells, rE2-200Q and rE2-200Q/E1-141Q retained WT virus-like adsorption efficiency. All other mutants exhibited reduced adsorption (*p* < 0.05). Entry capacities of mutants were comparable to WT virus, with minor non-significant reductions observed in some mutants (Fig 2a). These results are consistent with the trend of viral replication capability in BHK-21 cells; however, in C6/36 cells, rE1-141Q exhibited suppressed adsorption and entry. rE2-200Q and rE2-200Q/E1-141Q showed enhancement in both processes. rE2-262Q displayed impaired entry without affecting adsorption. rE2-262Q/E1-141Q exhibited increased capabilities in both adsorption and entry. rE2-200/262Q displayed reduced in both phases, while rE2E1Q showed no significant differences from WT virus (Fig 2b). In Vero cells, rE2-200/262Q and rE2E1Q retained WT virus-like binding capacity. Other mutants showed attenuated adsorption. During entry, only rE2E1Q matched WT virus efficiency, while others displayed reduced entry (*p* < 0.01; Fig 3a).

To define the basis for these altered attachment phenotypes, we assessed the interaction between glycosylation-deficient viruses and heparan sulfate (HS), a glycosaminoglycan (GAG), facilitates alphavirus attachment. First, we determined the non-toxic concentration of Hp for Vero cells and found that 20 mg/mL of Hp did not impair Vero cell viability (Fig 3a). To assess HS dependency, viruses were pre-incubated with heparin (Hp; 2 mg/mL) or PBS (control) and adsorbed to Vero cells at 4°C. RT-qPCR quantification revealed Hp reduced WT virus adsorption by ~10%. rE2-200Q, rE2-262Q, rE2-200/262Q, as well as rE2E1Q, exhibited greater inhibition (*p* < 0.05), indicating enhanced heparin-mediated inhibition and, by extension, a heightened dependency on cell-surface HS for attachment. Conversely, rE1-141Q showed enhanced adsorption post-Hp treatment (*p* < 0.001), suggesting a unique, glycan-dependent mechanism for this site. rE2-200Q/E1-141Q, rE2-262Q/E1-141Q showed no significant differences from WT virus (Fig 3b). Plaque assays corroborated these findings: Hp reduced WT virus plaque counts by ~30%, with stronger suppression observed for rE2-262Q (*p* < 0.01), rE2-200/262Q (*p* < 0.05), and rE2E1Q. rE1-141Q displayed increased plaque formation post-Hp treatment (*p* < 0.05; Fig 3c). These data suggest that N-glycosylation modulates the contribution of HS interactions during attachment.

To assess the impact of glycosylation mutations on virion assembly and infectivity of progeny virus, Vero cells were infected with WT virus or mutant viruses at a multiplicity of infection (MOI) of 5. After 1 hour of adsorption, unbound viruses were removed by washing three times with PBS. The infected cells were further incubated at 37°C, and both intracellular and extracellular levels of viral RNA and infectious virus were measured at 12 hpi and 18 hpi using RT-qPCR and TCID₅₀ assays, respectively.

To evaluate the efficiency of intracellular virion assembly, the intracellular viral RNA-to-TCID₅₀ ratio was calculated. At 12 hpi, the intracellular RNA/TCID₅₀ ratios of rE2-200Q, rE2-200Q/E1-141Q, and rE2E1Q were significantly higher than those of the WT virus (*p* < 0.05), suggesting impaired virion assembly efficiency. At 18 hpi, the elevated RNA/TCID₅₀ ratio of rE2-200Q/E1-141Q remained statistically significant (*p* < 0.05), further supporting reduced assembly efficiency. In contrast, the intracellular RNA/TCID₅₀ ratios of rE1-141Q, rE2-262Q, and rE2-262Q/E1-141Q were significantly lower than that of the WT virus (*p* < 0.05), indicating enhanced virion assembly efficiency (Fig 3b).

We next calculated the extracellular RNA/TCID₅₀ ratio to estimate the infectivity of progeny viruses released from Vero cells. As shown in Fig 3c, the extracellular RNA/TCID₅₀ ratios of WT virus were significantly higher than those of rE1-141Q, rE2-262Q, rE2-262Q/E1-141Q, and rE2-200/262Q at both 12 and 18 hpi (*p* < 0.05), indicating that WT progeny viruses were less infectious.

Furthermore, glycoprotein cleavage analysis determined by Western blot of Vero cells infected with WT virus or mutants confirmed intact cleavage of the E3-E2-6K-E1 polyprotein into pE2, E2, and E1 subunits. Anti-E2 and anti-E1 mAbs detected bands corresponding to each glycoprotein, indicating N-glycosylation deletions did not disrupt signal peptidase-mediated processing (Fig 2c).

In summary, although the removal of E protein glycosylation reduced viral attachment and entry in Vero cells, and altered viral dependency on heparan sulfate, it enhanced virion assembly efficiency and progeny virus infectivity under certain conditions, indicating that glycosylation differentially regulates distinct stages of the viral life cycle.

### 3.5. Reduction in disease severity of glycosylation-deficient virus-induced infection in 10-day-old mice

**Fig 4:**
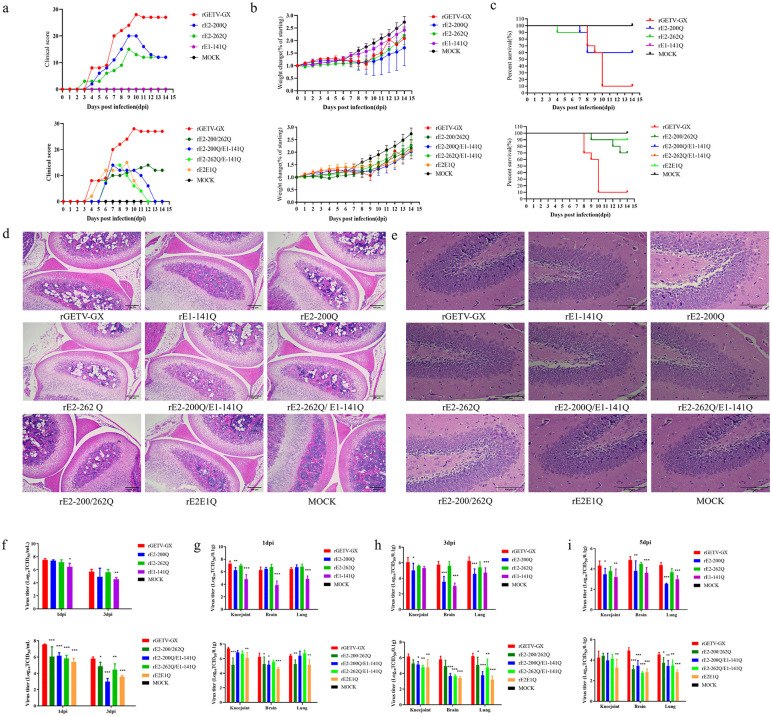
Pathogenicity of N-glycosylation-deficient viruses in neonatal and juvenile mice. 10-day-old **(a-c)** ICR mice were subcutaneously inoculated with 10⁴ TCID_50_ virus (DMEM-injected controls). Clinical scores **(a)**, body weight changes **(b)**, and survival rates (c) were monitored for 14 days. **(d-e)** Histopathological examination. Brain tissues and knee joints were fixed in 4% paraformaldehyde, trimmed, and paraffin-embedded (knee joints with prior decalcification). Sections (4-6 μm-thick) were mounted on slides and H&E-stained. **(d)** Knee joint **(e)** Brain tissue. (f-i) Viral replication kinetics in murine blood and tissues. Blood, knee joint, lung, and brain tissues were collected at 1, 3 and 5 dpi. Viral titers were determined by TCID_50_ assay. Data represent mean ± SD (n = 5; *p < 0.05; **p < 0.01; ***p < 0.001; Student’s *t*-test).

Having established that glycosylation mutants can replicate robustly in vitro, we asked whether this fitness translates to virulence in a mammalian host. We employed an age-dependent murine model to uncover virulence phenotypes that might be masked in highly susceptible neonatal hosts. 3- and 10-day-old mice were subcutaneously inoculated with 10^4^TCID_50_ of either WT virus or glycosylation-deficient mutant viruses and monitored for clinical outcomes. In 3-day-old mice, WT virus infection induced severe clinical manifestations, including markedly attenuated body weight gain relative to DMEM-inoculated controls (Fig 4a-b). While a single mouse infected with either the E1 (rE1-141Q) or E2 (rE2-200/262Q) glycosylation-deficient mutant survived, all other mutant-infected mice exhibited disease severity comparable to the WT virus group (Fig 4c).

In contrast, 10-day-old mice infected with WT virus displayed progressive weight loss, lethargy, hindlimb paralysis, and diarrhea by 5 dpi, with only one survivor (Fig 4a-c). Mice infected with glycosylation-deficient mutants exhibited delayed disease progression, entering a recovery phase 3–5 days after symptom onset despite initial clinical signs mirroring the WT virus group. Notably, the rE2-200Q demonstrated the lowest survival rate (60%) among glycosylation-deficient variants; however, this remained significantly higher than the WT virus group (Fig 4c). These findings indicate that ablation of envelope glycosylation attenuates viral pathogenicity in 10-day-old mice.

To determine the differences in pathological changes induced by mutant viruses versus the parental virus in mice, brain tissues and knee joints were collected from 10-day-old mice at 5 dpi for histopathological analysis. Compared to the negative control group, rGETV-GX-infected mice exhibited significant pathological alterations in knee joints, including reduced chondrocyte numbers, increased empty lacunae formed by clustered cells, severe cytoplasmic vacuolation, lysis and fragmentation of bone matrix cells with a significant numerical reduction, thinning and fragmentation of trabeculae, and increased bone porosity. Mice infected with rE1-141Q, rE2-200/262Q, and rE2E1Q showed milder knee joint pathology, whereas rE2-200Q and rE2-262Q caused more severe pathological lesions (Fig 4d). Pathological sections of brain tissues revealed varying degrees of degeneration and necrosis in cerebellar neurons of virus-infected mice, with nuclear pyknosis and dissolution, most pronounced in rGETV-GX-infected mice. In contrast, mutant viruses induced comparatively milder cerebral pathology (Fig 4e). These results demonstrate that the loss of glycosylation sites, particularly at the E1-N141 site, attenuates viral virulence in 10-day-old mice. The clear dissociation between efficient in vitro replication and attenuated in vivo virulence highlights the essential role of glycans in navigating the complexities of a living host.

### 3.6. Impaired viral replication in 10-day-old mice infected with glycosylation-deficient viruses

To assess whether reduced disease severity correlated with diminished viral replication, 3- and 10-day-old ICR mice were infected with WT virus or glycosylation-deficient viruses. Tissues (brain, lungs, knee joints) and serum were harvested at designated timepoints, and viral titers were quantified via TCID₅₀ assays.

In 3-day-old mice, serum viral loads in rE1-141Q and rE2-262Q infected groups exceeded WT virus levels at 1 dpi and 2 dpi, respectively. By 3 dpi, serum viral titers of single-glycosylation mutants (rE1-141Q, rE2-200Q or rE2-262Q) showed no significant difference from WT virus. However, rE2-200Q/E1-141Q exhibited significantly lower serum viral titers at 2 dpi and 3 dpi, while rE2E1Q produced markedly reduced viral titers relative to WT virus at 3 dpi (Fig 4d).

Tissue viral loads in single-glycosylation mutants were comparable to WT virus at 3 dpi, whereas rE2E1Q infection resulted in significantly attenuated tissue viral titers across all organs except knee joints at 3 dpi. Intriguingly, rE2-200/262Q and rE2-262Q/E1-141Q displayed elevated replication in knee joints relative to WT virus by 3 dpi (Fig 4e-g).

In 10-day-old mice, glycosylation-deficient mutants demonstrated enhanced viral clearance in serum by 1 dpi and 3 dpi, except for rE2-200Q and rE2-262Q (Fig 4f). rE1-141Q exhibited significantly reduced tissue titers across all organs except knee joints at 3 dpi, while rE2-200Q-infected mice showed diminished viral loads in tissues by 3–5 dpi. rE2-200/262Q displayed transient replication in knee joints, peaking at 3 dpi but remaining below WT virus levels. rE2E1Q exhibited universally attenuated viral burden across tissues, including suppressed serum viremia (Fig 4g-i). rE2-200/262Q, rE2-200Q/E1-141Q and rE2-262Q/E1-141Q showed compartment-specific suppression, with brain and lung titers consistently reduced relative to WT virus at 3 dpi and 5 dpi. These data unequivocally demonstrate that the loss of envelope glycans significantly compromises the ability of GETV to establish and maintain a robust infection in juvenile mice. This directly links the reduced in vivo replication efficiency to the observed attenuation in pathogenicity, underscoring the critical role of glycosylation in viral fitness within an immunocompetent host.

### 3.7. Viral replication kinetics in mosquito vectors

**Fig 5:**
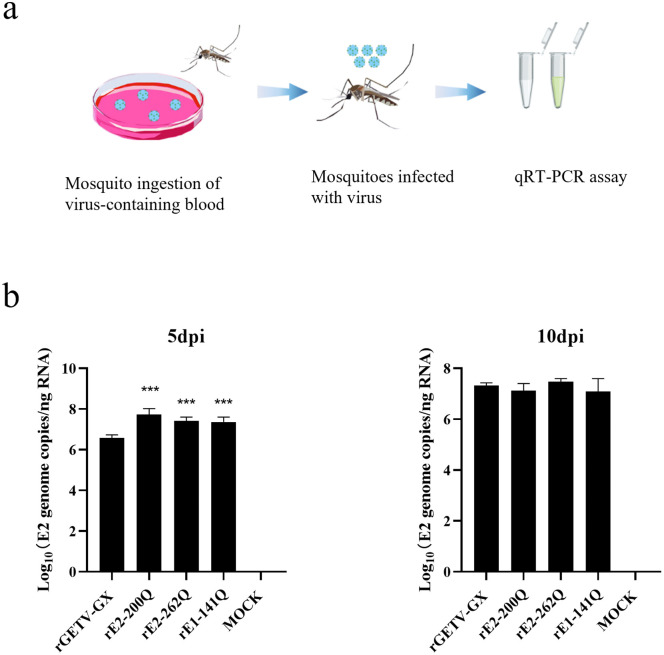
Replication competence of glycosylation-deficient viruses in mosquitoes. **(a)** *Aedes albopictus* mosquitoes were fed virus mixed with defibrinated sheep blood and 10% sucrose. **(b)** Viral RNA copies in whole mosquitoes (5 and 10 dpi) were quantified by RT-qPCR.

Based on the paradoxical phenomenon observed in mammalian models where deletion of glycosylation sites enhances viral replication in vitro but attenuates virulence in vivo, we focused on two key glycosylation sites, E2-N262 and E1-N141, to investigate whether their absence balances interhost infection and transmission by altering viral fitness in mosquito vectors. To assess the impact of glycosylation site deletions on arthropod replication, mosquitoes were fed blood meals containing equivalent titers of WT virus or mutant viruses (rE2-200Q, rE2-262Q and rE1-141Q) (Fig 5a). At 5 dpi, mutant viruses demonstrated enhanced replication efficiency in mosquitoes relative to WT virus. However, by 10 dpi, replication levels of all mutants converged with WT virus titers (Fig 5b), suggesting transient fitness advantages for glycosylation-deficient variants in the invertebrate host, while maintaining long-term fitness comparable to wild-type virus. This transient boost, coupled with mammalian attenuation, suggests a complex trade-off where glycans fine-tune viral fitness differently in each host to optimize overall transmission efficiency.

### 3.8. Enhanced susceptibility of glycosylation-deficient viruses to neutralizing antibodies

**Fig 6:**
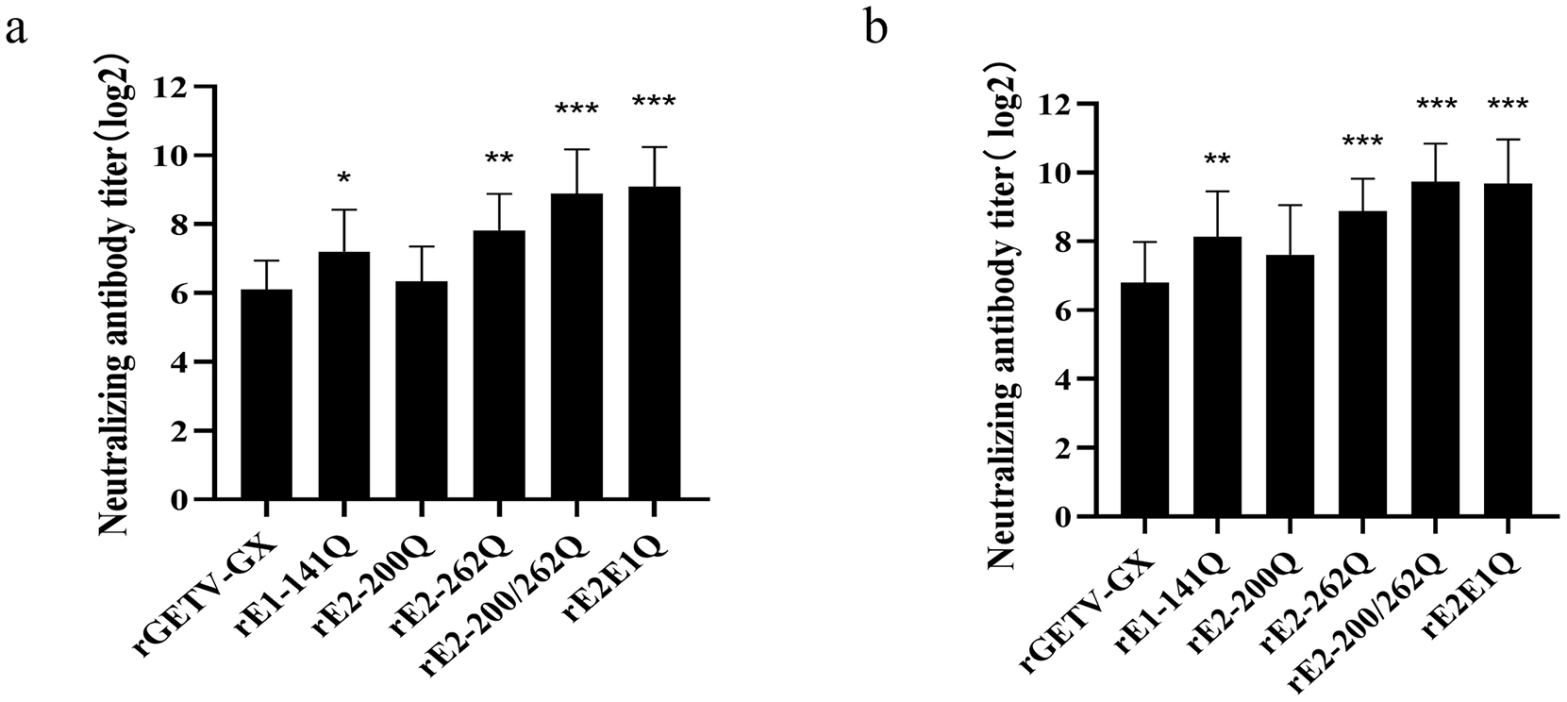
Neutralizing antibody sensitivity of glycosylation-deficient viruses. Viruses (200 TCID_50_) were incubated with serially diluted inactivated sera (37°C, 2 hours) and added to 96-well cell cultures. Post-adsorption (37°C, 1 hour), cells were washed and maintained in 2% DMEM. CPE-based neutralizing titers were calculated for (a) swine GETV-positive serum and (b) murine GETV-positive serum.

Following the demonstration of an attenuated phenotype for glycosylation-deficient viruses in the mouse model, we further investigated how the loss of glycans affects their interaction with the humoral immune response. A canonical function of viral envelope glycosylation is to mediate immune evasion by shielding underlying protein epitopes from antibody recognition. We therefore hypothesized that the removal of these glycans would expose conserved neutralizing epitopes on the GETV surface, thereby increasing the virus’s sensitivity to the humoral immune response. Neutralization assays were performed to compare the sensitivity of WT virus and glycosylation-deficient mutants to neutralizing antibodies (NAb). Sixteen porcine-derived clinical serum samples confirmed as GETV antibody-positive were analyzed. Deletion of the E2-200 glycosylation site elevated neutralizing antibody titers in a subset of sera with no statistically significant difference, whereas ablation of the E2-262 site significantly enhanced neutralization titers across all sera (*p* < 0.01). A more pronounced increase in neutralizing activity was observed for mutants lacking double and triple glycosylation sites. Deletion of the E1-141 glycosylation site similarly enhanced neutralization titers (Fig 6a). Parallel experiments using murine GETV-positive antisera corroborated these findings, with double or triple glycosylation-deficient mutants eliciting the highest neutralizing antibody responses (Fig 6b). This provides direct functional evidence that N-glycans on the GETV envelope, particularly the epidemic-lineage-specific E2-N262 glycan, facilitate immune evasion by acting as a “glycan shield”. This finding mechanistically explains one key evolutionary advantage for the acquisition and retention of the E2-N262 site in circulating strains, as it directly enhances the virus’s ability to circumvent host neutralizing antibodies, a critical factor for sustained transmission and outbreak potential.

### 3.9. Impact of N-glycosylation site deletions on neutralizing antibody responses in vivo

Given that glycan removal increases viral susceptibility to NAbs, we next examined whether these less shielded variants might also elicit a more robust NAb response upon immunization, with important implications for vaccine development. To evaluate the role of N-glycosylation in eliciting neutralizing antibody responses, six groups of 6-week-old female ICR mice were immunized with WT virus or glycosylation-deficient viruses. Homologous neutralization titers (against the immunizing virus) were assessed in sera collected at 14–35 dpi. Mice inoculated with rE2-200/262Q or rE2E1Q exhibited earlier and significantly elevated homologous NAb titers relative to WT-virus infected mice at 14–28 dpi (Table 1). Although rE1-141Q infection induced higher neutralization titers than WT virus, statistically significant differences were observed only at 21 dpi. rE2-200Q or rE2-262Q did not differ significantly from WT virus in homologous NAb induction.

**Table 1:** Impact of N-glycosylation on neutralizing antibody induction.

Inoculated strain	Neutralizing antibody titers measured against homologous strains^a^			
	14 dpi	21 dpi	28 dpi	35 dpi
rGETV-GX	5.67 ± 0.22	6.59 ± 0.75	6.58 ± 0.21	6.40 ± 0.3
rE1-141Q	6.32 ± 0.12	7.51 ± 0.67^**^	8.11 ± 0.54	5.43 ± 0.3
rE2-200Q	4.80 ± 0.35	4.85 ± 0.56	6.19 ± 1.11	5.97 ± 1.08
rE2-262Q	6.37 ± 1.57	5.07 ± 0.44	4.75 ± 0.15	7.49 ± 0.62
rE2-200/262Q	9.21 ± 0.88^***^	10.20 ± 0.79^***^	9.79 ± 0.16^***^	5.54 ± 0.15
rE2E1Q	9.67 ± 0.31^***^	8.77 ± 1.39^*^	10.22 ± 0.08^***^	6.31 ± 0.84

Neutralizing antibody responses induced by glycosylation-deficient viruses. 6-week-old mice were injected with 10⁴ TCID_50_ virus. Sera collected at indicated time points were tested for neutralization against homologous virus. a: Neutralization titers are expressed as the reciprocal of the highest dilution and were log2-transformed. Data represent mean ± SD. (n = 3; *p < 0.05; **p < 0.01; ***p < 0.001, Student’s *t*-test).

To assess cross-neutralization against the fully glycosylated strain rGETV-GX, antisera from mutants-infected mice showed reduced neutralizing activity against rGETV-GX compared to WT virus antisera, though differences were not statistically significant. Notably, antisera from rE2-200/262Q or rE2E1Q achieved cross-neutralization titers comparable to those of WT virus antisera (Table 2). Our results demonstrate that the acquisition of the E2-N262 glycan in epidemic lineages serves as a potent glycan shield, and its ablation tilts the balance away from immune evasion, despite the potential replicative benefits observed in Fig 3. These results reveal the combined, but not individual, ablation of E2 glycosylation sites induces robust early homologous neutralizing antibody responses while largely preserving cross-neutralization capacity. This finding is crucial for vaccine design, suggesting that strategic glycan removal can modulate immunogenicity, but its utility for generating broad protection may be limited.

**Table 2:** Neutralizing activities of serum samples measured against WT virus.

Inoculated strain	Neutralizing antibody titer^a^			
	14 dpi	21 dpi	28 dpi	35 dpi
rGETV-GX	5.67 ± 0.22	6.59 ± 0.75	6.58 ± 0.21	6.40 ± 0.3
rE1-141Q	5.46 ± 0	5.85 ± 0.43	6.25 ± 0.21	5.83 ± 0.27
rE2-200Q	5.15 ± 0.49	4.46 ± 0	5.52 ± 0.09	5.58 ± 0.7
rE2-262Q	4.49 ± 0.16	5.53 ± 0.57	5.00 ± 0.4	4.68 ± 0.5
rE2-200/262Q	6.50 ± 1.32	7.41 ± 0.88	6.11 ± 0.44	5.83 ± 0.27
rE2E1Q	5.97 ± 0.27	6.31 ± 0.27	5.81 ± 0.35	6.33 ± 0.11

Neutralizing antibody responses induced by glycosylation-deficient viruses. 6-week-old mice were injected with 10⁴ TCID_50_ virus. Sera collected at indicated time points were tested for neutralization against WT virus. a: Neutralization titers are expressed as the reciprocal of the highest dilution and were log2-transformed. Data represent mean ± SD.

### 3.10. In vivo selection pressures drive glycosylation site restoration

To evaluate the genetic stability of glycosylation-deficient viruses in 6-week-old female murine infection models, serial blood samples were collected at 3, 7, and 14 dpi for RNA isolation. Circulating viral RNA copy numbers were quantified via RT-qPCR, while sequences encoding the E protein were amplified, cloned into T-vectors, and subjected to Sanger sequencing. Experimental findings demonstrated that mutant viruses deficient in specific glycosylation motifs exhibited moderately elevated viral RNA loads relative to WT virus controls, though these disparities lacked statistical significance. Notably, by 14 dpi, rE2-200/262Q and rE2E1Q displayed significantly higher viral titers than WT virus counterparts, with statistically robust differences (*P* < 0.05) observed exclusively in the rE2E1Q cohort (Fig 5). This outcome stands in contrast to prior observations of high-titer homologous NAbs in murine sera by 14 dpi.

To elucidate this discrepancy, Sanger sequencing was conducted to analyze the viral E protein sequences. Sequencing of rE2-200/262Q and rE2E1Q revealed partial restoration of N-glycosylation motifs at E2 position 200 at 7 dpi, while complete reversion to the canonical glycosylation sequence at E2 position 262 was observed in all analyzed samples. By 14 dpi, rE2E1Q demonstrated complete restoration of both glycosylation motifs across all specimens, whereas rE2-200/262Q retained partial mutation at the E2 200 position. Single-site E2 glycosylation mutants (rE2-262Q) exhibited progressive motif restoration starting at 7 dpi and achieving full reversion to native sequences by 14 dpi, while the E2-200 locus always maintained the mutated motifs. In contrast, E1 glycosylation deletions remained genetically stable across all mutants (Table 3). Notably, recurrent amino acid substitutions were identified in E2 across multiple mutants (Table 4). This provides direct in vivo evidence of an immense evolutionary selection pressure to maintain glycosylation, especially at the E2-N262 site. This pressure likely reflects the combined advantage of glycan shielding for immune evasion and optimal receptor interactions for infectivity, underscoring the critical fitness cost associated with the loss of this epidemic-lineage-specific glycan.

**Table 3:** Stability analysis of glycosylation-deficient viruses in vivo.

Inoculated strain	Amino acid change (N)/Original amino acid (Q)					
	7 dpi	7 dpi	7 dpi	14 dpi	14 dpi	14 dpi
	N141Q	N200Q	N262Q	N141Q	N200Q	N262Q
rE1-141Q	0/3	/	/	0/2	/	/
rE2-200Q	/	1/2	/	/	3/3	/
rE2-262Q	/	/	1/2	/	/	3/3
rE2-200/262Q	/	1/4	4/4	/	4/5	5/5
rE2E1Q	0/3	1/3	3/3	0/2	3/3	3/3

Genetic stability and reversion analysis of glycosylation-deficient viruses. E1 and E2 genes from infected tissues were amplified by RT-PCR, cloned into a TA vector, and sequenced. Percentages indicate variants reverting to wild-type glycosylation motifs.

**Table 4:** Additional amino acid alterations in the E protein of the mutant virus.

Strain	Gene names	The amino acid position	Original amino acid/ Amino acid change
rE2-200Q	E2	209	G/D
rE2-262Q	E2	214	S/C
rE2-262Q	E2	258	F/S
rE2-200/262Q	E2	150	E/G
rE2-200/262Q	E2	218	T/K
rE2-200/262Q	E2	239	S/T
rE2-200/262Q	E2	269	V/L
rE2E1Q	E2	151	V/A
rE2E1Q	E2	153	C/R
rE2E1Q	E2	157	Q/R
rE2E1Q	E2	185	S/P
rE2E1Q	E2	201	C/Y
rE2E1Q	E2	212	C/R
rE2E1Q	E2	213	S/R
rE2E1Q	E2	223	A/V
rE2E1Q	E2	279	K/E
rE1-141Q	E1	215	A/V
rE1-141Q	E1	279	S/P
rE1-141Q	E1	293	A/V
rE1-141Q	E1	315	S/I
rE1-141Q	E1	318	L/P
rE2E1Q	E1	158	G/D
rE2E1Q	E1	218	A/T
rE2E1Q	E1	296	V/I
rE2E1Q	E1	304	A/V

### 3.11. Structural modeling and SPR experiments show that glycosylation modifies virus-host interactions

**Fig 7:**
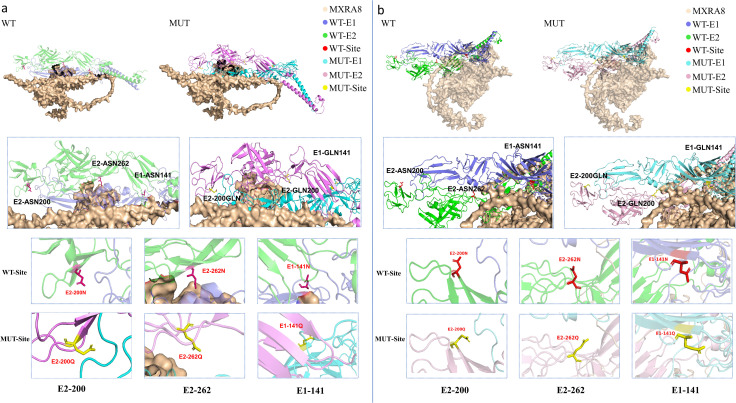
Prediction of the E1-E2 and MXRA8/LDLR Protein Binding Model. Three-dimensional model of E1-E2, constructed by homology modeling using cryo-EM structure of GETV (PDB: 7WC2) as the template. The constructed E1-E2 model was used as a ligand and docked with the structures of the receptor proteins MXRA8 (UniProtKB: Q9DBV4) and LDLR (UniProtKB: P35951) via the GRAMM web server. The docking results were visualized and the interaction interfaces were analyzed using PyMOL and PDBePISA. **(a-b)** Schematic representation of the deglycosylated amino acid sites in E1-E2 and the predicted interaction forces with the MXRA8 protein (a) and the LDLR protein (b).

To elucidate the structural basis of glycosylation-mediated functions, we performed homology modeling of the GETV E1-E2 heterodimer using the SWISS-MODEL server with Cryo-EM structure of GETV (PDB: 7WC2) as a reference. Subsequent molecular docking analyses with two established alphavirus receptors, MXRA8 and LDLR, yielded distinct insights. Docking of a full-length E1-E2 dimer model with the MXRA8 receptor revealed a glycan-sensitive interaction landscape. Local structural analysis of the three N → Q substitutions reveals that the mutations primarily remodel the MXRA8-proximal surface without perturbing the global fold. Ablation of glycans at both E1-N141 and E2-N262 was accompanied by a redistributed interaction network and, in the docked poses, a higher number of putative hydrogen bonds, suggesting a potential alteration in the receptor engagement mechanism. Conversely, the removal of the E2-N200 glycan led to a loss of pre-existing hydrogen bonds, indicating its role in stabilizing the wild-type interaction with MXRA8. Specifically, the E2-N200Q substitution extends the side chain and reorients the terminal amide toward the solvent, thereby rewiring local hydrogen-bond geometry and destabilizing the original interaction network at the contact surface. In E2-N262Q, the longer Gln side chain is accompanied by subtle loop relaxation, which weakens packing against the adjacent β-strand and reduces local shape complementarity with the receptor. For E1-N141Q, the shift of the side chain toward the MXRA8-facing surface introduces steric bulk and alters the electrostatic character at the edge of the binding pocket, likely affecting encounter-complex formation and productive engagement (Fig 7a). Collectively, the mutations do not disrupt the core secondary structure (β-sheets and α-helices) but significantly alter the geometric features and polar complementarity of the binding pocket, thereby providing a structural basis for the observed functional changes. In contrast, docking with the predicted structure of LDLR revealed that regardless of their glycosylation status, none of the three sites (E1-N141, E2-N200, or E2-N262) formed direct hydrogen bonds with LDLR, suggesting these specific glycans are not directly involved in LDLR binding (Fig 7b). However, all three glycosylation sites were located within the predicted LDLR binding interface, implying a potential for indirect modulation of the interaction.

**Fig 8:**
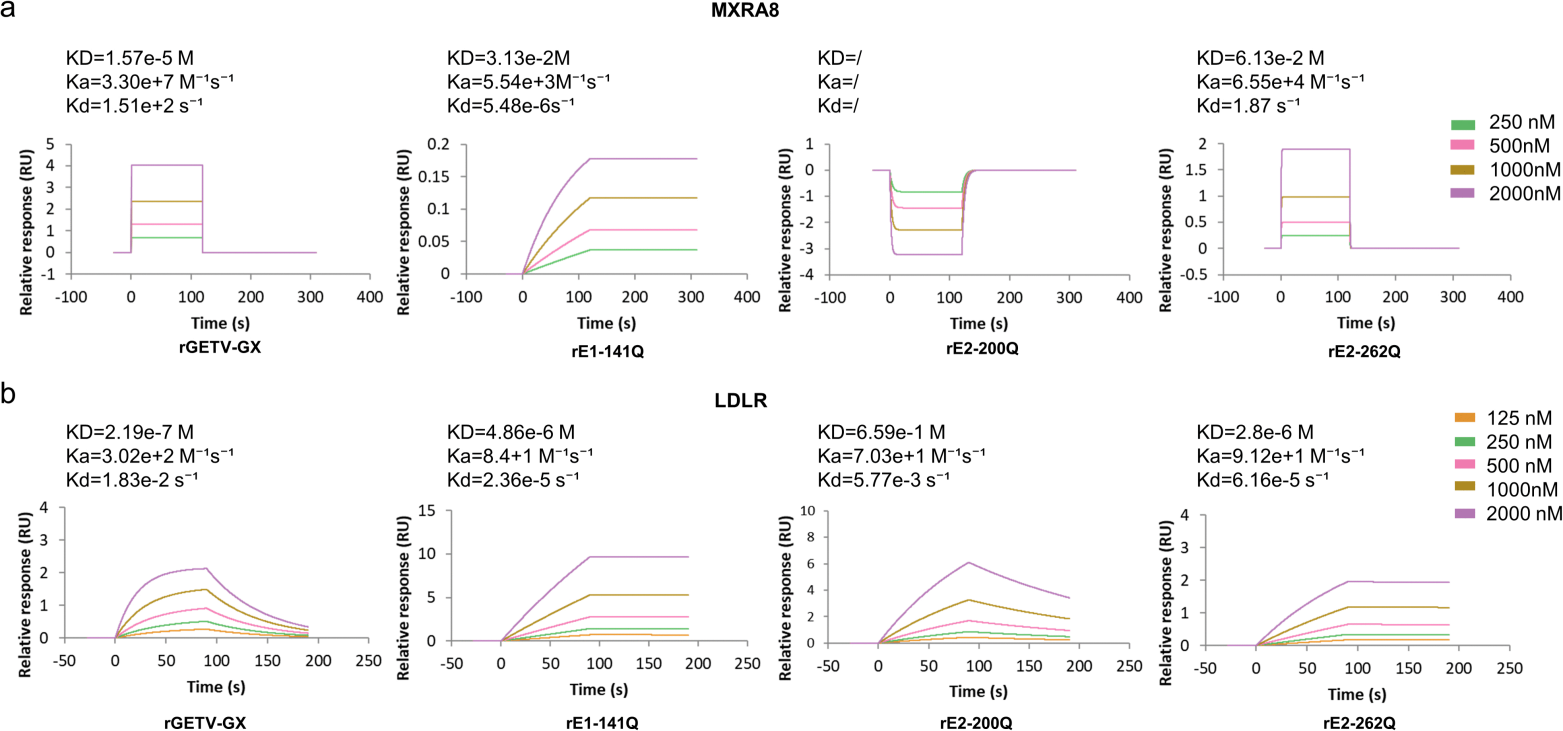
SPR analysis of binding between purified GETV particles and MXRA8/LDLR. **(a)** Representative SPR sensorgrams showing binding of purified rGETV-GX, rE1-141Q, rE2-200Q, and rE2-262Q virions to immobilized MXRA8. Virions were injected at serial concentrations. Apparent affinities were obtained by fitting with a 1:1 Langmuir model. rE2-200Q showed no detectable binding to MXRA8 under the tested conditions. **(b)** Representative SPR sensorgrams showing binding of purified virions to immobilized LDLR at the indicated concentrations. Apparent affinities were obtained as in **(a)**.

To obtain direct binding evidence for glycan-dependent engagement of host factors, we performed SPR assays using purified GETV particles as analytes and immobilized MXRA8 or LDLR as ligands (Fig 8a-b). WT virus showed measurable binding to both MXRA8 and LDLR. In contrast, all three deglycosylation mutants exhibited reduced binding responses and lower apparent affinity compared with WT. For MXRA8, rE1-141Q and rE2-262Q bound weakly, whereas rE2-200Q produced no detectable binding signal under the tested conditions (Fig 8a). For LDLR, all three mutants showed reduced binding relative to WT, with rE2-200Q displaying the strongest reduction (Fig 8b).

Because whole virions were used as analytes and parameters were derived from a single global fit of a representative dataset, the results are interpreted as apparent affinities that may incorporate avidity and model-dependent effects. Nevertheless, the consistent reduction in binding upon glycan ablation supports a functional contribution of envelope N-glycans to MXRA8 engagement and to measurable LDLR binding in vitro.

## 4.  Discussion

Viral envelope protein N-linked glycosylation plays a pivotal role in modulating host-pathogen interactions, influencing glycoprotein folding, trafficking, stability, virion assembly, immune evasion, and pathogenicity [42]. While cryo-electron microscopy (cryoEM) has identified surface-exposed N-glycosylation sites near GETV E1/E2 trimer interfaces [43,44], their functional contributions in GETV remained incompletely defined. This study investigated site-specific N-glycosylation effects on GETV fitness via seven glycosylation-deficient mutants, revealing these glycans are not just structural but critical for tuning viral replication, host tropism, virulence, and immune evasion—with evolution favoring their retention to optimize epidemic fitness. These conclusions are supported by a recent study identifying E2-N262 glycosylation as a key determinant of GETV mammalian pathogenesis and host adaptation [45]. Notably, ablation of N-glycosylation at individual or combined sites did not impair viral rescue or polyprotein processing, consistent with Semliki Forest virus (SFV) findings where glycosylation modifications were dispensable for pE2 cleavage [46]. Multi-step growth kinetics showed cell type- and glycosylation site-dependent replication effects, contrasting with Sindbis virus (SINV, E1 glycosylation deletion attenuates vertebrate cell replication [47]), and Salmonid Alphavirus (SAV, requires E1 glycosylation for virion production [48]). This divergence highlights E1 glycosylation functional plasticity across alphaviruses, likely reflecting host niche adaptations.

Glycosylation absence differentially impacts viral infectivity and replication in vitro and in vivo. Guided by high-resolution cryo-EM, we found site-specific N-glycans balance viral entry and stability. Surface-exposed glycans at E1-N141, E2-N200, and E2-N262 locate at key functional interfaces: epidemic-lineage-specific E2-N262 (E2 domain B near receptor-binding “canyon”) forms a “handshake” with adjacent spike glycans, restricting spike flexibility and receptor access; highly conserved E1-N141 (E1 domain I β-hairpin tip) interacts with MXRA8 in related alphaviruses; E2-N200 (E2 domain B tip) forms hydrogen bonds with E1 fusion loop E99 to stabilize structure [43, 44]. Removing E2-N262 and E1-N141 glycans may induce a degree of “conformational release,” thereby altering E1–E2 spike dynamics and the presentation of receptor-/attachment-factor–interacting surfaces. Molecular docking predicts remodeled MXRA8-binding interfaces upon glycan ablation; however, our SPR measurements indicate that glycan removal reduces measurable binding to MXRA8 in vitro, suggesting that increased surface exposure alone may be insufficient to enhance binding and may occur alongside reduced spike stability and/or shifts in the population of binding-competent conformations.

During alphavirus assembly, structural polyproteins are cleaved to release the capsids, while the p62-6K-E1 precursor translocates to the endoplasmic reticulum (ER) for post-translational modifications (glycosylation, disulfide bond formation [49]). E1/E2 glycosylation absence may disrupt ER processing of this precursor, destabilizing the E1-E2 heterodimer (critical for spike assembly and fusogenicity)—aligning with Chikungunya virus (CHIKV) studies, where E1 hinge/ domain II perturbations alter E2 conformation, receptor engagement, and infectivity [50].

Notably, combined glycan ablation disrupts virion assembly and reduces particle fitness, evidenced by the triple mutant’s high intracellular RNA-to-infectivity ratio. The GETV structure also identifies a hydrophobic pocket between E1/E2 transmembrane helices, stabilized by cholesterol and phospholipids (e.g., DOPC). Though not directly glycosylated, this pocket’s stability depends on ectodomain conformation, so glycan-mediated E2 changes may exert long-range effects on pocket and envelope membrane stability.

Our functional studies show glycosylation ablation has complex effects on GETV attachment and entry. Consistent with our SPR measurements, glycan ablation reduced measurable binding to MXRA8 and LDLR in vitro, with the most pronounced impairment observed for rE2-200Q. We note that the reported receptor preferences of E2-N262 glycosylation can vary across viral backbones, glycan-ablating substitutions (e.g., N262D versus N262Q), and binding platforms (whole-virion SPR versus VLP/ectodomain-based assays) [45]. Molecular docking provides potential structural mechanisms: while studied glycosylation sites do not form direct hydrogen bonds with LDLR, they lie within predicted binding interfaces and may modulate binding via allosteric effects. More critically, MXRA8 docking indicates E1-N141/E2-N262 glycan removal remodels the binding interface and alters hydrogen-bond networks—providing possible structural hypotheses for the observed entry phenotypes. However, the precise structural/mechanistic basis for these differences remains unclear and requires further study.

Beyond entry, glycosylation impacts late viral life cycle stages. High intracellular viral RNA but low infectious titers (e.g., rE2E1Q, rE2-200Q/E1-141Q) indicate virion assembly defects or poorly infectious particles. Glycans facilitate ER glycoprotein folding [21], so their absence likely perturbs E1-E2 heterodimer folding/stability, reducing infectious virion assembly. This “assembly inefficiency” is a potentially underappreciated GETV envelope glycosylation role and likely contributes to in vivo attenuation. Conversely, rE1-141Q and rE2-262Q’s improved Vero cell assembly efficiency shows glycan removal can optimize infectious progeny production in specific scenarios, underscoring site-specific effects.

Integrating these data, we unify these findings in a hypothesis: GETV envelope site-specific N-glycosylation is an evolutionary adaptation that optimizes viral fitness by balancing replication efficiency, particle fitness/assembly, and immune evasion. Strong in vivo selection for E2 glycosylation mutant reversion—especially at E2-N262 (a Group III (GIII) epidemic-lineage marker)—highlights this balance’s criticality. Though E2-N262 may not be the most critical factor in individual assays (e.g., replication, mosquito propagation), its consistent reversion indicates it optimizes overall fitness in mammalian hosts (where immune pressure is paramount). Its lineage-specific acquisition likely expanded GIII GETV’s host tropism and epizootic potential.

Arboviruses propagated in mammalian versus invertebrate cells exhibit divergent receptor specificities [51]. Heparan sulfate (HS), a ubiquitous glycosaminoglycan, is an alphavirus attachment factor. Our data show ablating individual E2 glycosylation sites reduces viral adsorption to mammalian cells, likely reflecting the combined effects of impaired specific receptor engagement (consistent with reduced MXRA8/LDLR binding in SPR) and altered attachment-factor usage. Notably, heparin inhibition was enhanced for several glycosylation-deficient mutants (e.g., E2-N262Q), suggesting increased functional reliance on HS/GAG-mediated attachment during entry rather than unequivocally increased HS affinity. These seemingly divergent phenotypes support a model in which glycan loss reshapes the balance between specific receptor engagement and GAG-dependent attachment, potentially through changes in surface charge distribution and/or exposure of cryptic HS-interacting regions.

Notably, alphavirus entry is not exclusively GAG-dependent. CHIKV replicon particles can infect GAG-negative cells [52], and some strains utilize clathrin-independent endocytosis or direct membrane fusion [53]. Glycosylation-deficient mutants inhibitor sensitivity may reflect altered virion charge distribution or conformational changes that unmask cryptic receptor-binding domains. Recent structural studies show E1’s unexpected receptor role: human MXRA8 R242 interacts with the E1-N141’s N-acetylglucosamine (NAG) [34], challenging the canonical view of E2 as the sole mediator of attachment and highlighting the need to reevaluate glycosylation’s role in spike-receptor crosstalk.

N-glycosylation is a key alphavirus pathogenicity determinant [54]. Age-dependent alphaviral attenuation is well-documented [55], e.g., SINV kills neonatal mice at a dose of 1 PFU, but not adult mice [56], and juvenile mice are more vulnerable to SINV encephalomyelitis [57]. Our glycosylation-deficient mutants’ severe attenuation in 10-day-old mice (with more competent innate immunity than 3-day-old neonates) suggests enhanced innate immune recognition/clearance. Viral envelope glycosylation profoundly influences innate immune recognition: e.g., Ross River virus (RRV) N-glycans modulate type I interferon production in myeloid dendritic cells, and mosquito-derived alphaviruses (bearing distinct glycan profiles) evade innate immune recognition compared with their mammalian-derived counterparts [33]. Specifically, mammalian-derived RRV (mam-RRV, enriched in complex N- glycans), triggers strong type I interferon (IFN) responses, while mosquito-derived RRV (mos-RRV, with high-mannose/paucimannose oligosaccharides), evades myeloid cell recognition [58]. Envelope glycosylation also governs cutaneous infection immune modulation and pathogenesis [59]. Glycan ablation may expose conserved viral epitopes, increasing pattern recognition receptor or neutralizing antibody recognition. Alternatively, deglycosylation-induced altered receptor usage/tropism may affect innate immune cell infection/replication. While our data confirm glycosylation deletions impair GETV in vivo fitness, defining how these modifications interact with/evade the innate immune system remains a key future direction.

HS binding and virulence have a multifaceted relationship. In vertebrates, rapid viral replication drives high-titer viremia (required for systemic dissemination), but HS binding may attenuate diseases by promoting hepatic clearance of circulating virions [60], reducing viremic magnitude/duration and limiting cutaneous dissemination/neuroinvasion [61]. Conversely, strong HS binding may amplify local pathogenicity by attaching to HS-rich tissues (e.g., central nervous system [62]). Host-specific glycosylation complicates this: divergent N-glycan processing in mosquitoes vs. mammalian generates glycoproteins with distinct receptor affinities and virulence profiles. Our experiments show that glycosylation-deficient viruses reach early viremic peaks comparable to WT virus but clear faster—potentially due to altered GAG interactions or enhanced immune recognition.

Cross-species arboviral transmission often relies on single-nucleotide polymorphisms (SNPs) that enhance host adaptation [63,64]. Wild boars, horses, and swine are putative GETV reservoirs [35], but epizootics require competent mosquito vectors. Among the four GETV lineages, GIII strains have broad vector diversity, expanded host tropism, and exclusive epizootic association—traits linked to a novel E2-N262 N-glycosylation motif (absent in prototype Group I (GI) strains, present in GII/GIII/GIV).

To assess E2-N262’s role in vector competence, *Aedes albopictus* mosquitoes were orally challenged with isogenic glycosylation-deficient mutants and WT viruses. Both variants and WT virus had comparable replication efficiency [65], though mutants showed faster early replication before eventually reaching WT strain titers. This suggests glycosylation does not impair midgut infection or salivary gland escape—key arboviral transmission bottlenecks [66]. Further studies should explore how glycan-mediated structural changes modulate lineage-specific adaptations in vector tropism, replication, and interhost transmission.

Viral glycosylation canonical function involves immune evasion via glycan shielding of immunogenic epitopes (conserved in coronaviruses, HIV-1, influenza). GETV infection induces rapid neutralizing antibody (NAb) production [67]. Our data show glycosylation ablation—especially E2-N200/E2-N262 dual deletion—significantly increases viral susceptibility to NAbs. E2-N262 (acquired in later GETV lineages) may be an immune evasion adaptation, while conserved E1-N141/E2-N200 glycosylation is critical for arthritogenic strain immune resistance. Together, these observations suggest that GETV envelope glycosylation promotes antibody evasion in our experimental setting, consistent with a glycan-shielding mechanism.

Notably, glycan removal can paradoxically enhance immunogenicity: carbohydrate-depleted envelope glycoproteins increase NAb titers against mutants and sometimes cross-reactive responses against WT virus [68–70]. To assess N-glycosylation’s role in NAb induction, mice were infected with glycosylation-deficient mutants. E2 single-glycan mutants did not enhance NAb responses, but E1 glycosylation deletion significantly increases homologous NAb titers by 21 dpi (Table 1). Early rE2-200/262Q and rE2E1Q infection elicited stronger homologous NAbs, though WT virus cross-neutralization remained unchanged (Table 2). We hypothesize host immune pressure drives rapid glycan restoration at depleted sites, preventing sustained anti-WT virus responses—supported by observed E2 β-ribbon domain compensatory mutations (e.g., residue 269, linked to E1 binding, antibody evasion, and receptor interaction [43,71]). Though this site is under positive selection [35,72], partial reversion to ancestral residues in our sequencing data suggests a balance between infectivity optimization and immune escape, requiring functional validation.

In conclusion, we propose that GETV E1/E2 N-glycans act as integrated determinants of viral fitness by stabilizing receptor-binding-competent spike conformations, tuning the relative contributions of MXRA8/LDLR engagement versus GAG-mediated attachment, and masking neutralizing epitopes. This model reconciles the context-dependent in vitro phenotypes with the reduced net virion–receptor binding measured by SPR and the increased neutralization sensitivity observed upon glycan ablation. Consistent with this framework, rapid in vivo reversion of E2 glycosylation—most prominently at the epidemic-lineage-associated E2-N262 site—highlights strong host-imposed selection to maintain glycan-dependent fitness, revealing an evolutionary trade-off that balances replication/particle output, mosquito transmission, mammalian virulence, and immune evasion across hosts. Defining how glycosylation-driven conformational dynamics mechanistically couple receptor usage to antibody recognition will be important for rational vaccine design and antiviral development.

## Supporting information

S1 Fig Prediction of glycosylation sites in the GETV E protein and identification of mutant viruses. (a) Potential N-glycosylation motifs in E1 and E2 glycoproteins were computationally predicted using the NetNGlyc 1.0 server. (b) CPE induced by mutant viruses in BHK-21 cells. (c) Sanger sequencing of the rescued P3 virus. (d) Western blot analysis of glycosylation status. Third-passage (P3) viral lysates were clarified by centrifugation (15,000 × g, 4°C), resolved by SDS-PAGE, and immunoblotted with E2 mAb, E1 mAb, or capsid monoclonal antibody (Cap mAb). Migration patterns of E1, E2, and Cap proteins were compared. HRP-conjugated goat anti-mouse IgG served as the secondary antibody.
(TIF)

S2 Fig The impact of glycosylation site removal on viral adsorption/entry and polyprotein cleavage. (a-b) Attachment (4°C, 1 hour) and entry (37°C/28°C, 1 hour) efficiencies of WT virus and mutant viruses in cells (MOI = 5). Viral genome copies were quantified by RT-qPCR. (c) Western blot analysis of E1 and E2 cleavage kinetics. Vero cells infected with WT virus or mutants (MOI = 1) were lysed at 12, 18, and 24 hpi. Protein bands were detected using E1- or E2-specific mAbs.
(TIF)

S3 Fig Heparin (Hp) inhibition of viral attachment. (a) Cytotoxicity of Hp in Vero cells assessed via CCK-8 assay after 24-hour exposure. (b) Hp binding inhibition: Viruses (MOI = 5) pre-incubated with 2 mg/mL Hp (37°C, 1 hour) were adsorbed to cells (4°C, 1 hour). Cell-associated viral RNA was quantified by RT-qPCR. (c) Plaque reduction assay with 0.2 mg/mL Hp (MOI = 0.01). Plaque counts normalized to untreated controls. Statistical significance: *p < 0.05; **p < 0.01; ***p < 0.001 (Student’s *t*-test).
(TIF)

S4 Fig 3-day-old ICR mice were subcutaneously inoculated with 10⁴ TCID_50_ virus. Clinical scores (a), body weight changes (b), and survival rates (c) were monitored for 14 days. (d-g) Viral replication kinetics in murine blood and tissues. Viral titers were determined by TCID_50_ assay. Data represent mean ± SD (n = 5; *p < 0.05; **p < 0.01; ***p < 0.001, Student’s *t*-test).
(TIF)

S5 Fig Viral replication kinetics in six-month-old mice blood. Six-month-old SPF ICR mice were injected with 10⁴ TCID_50_ of the virus. Blood samples were collected at 3, 7, and 14 dpi. Viral RNA copies in serum were quantified by RT-qPCR. Data represent mean ± SD (n = 3; *p < 0.05; **p < 0.01; ***p < 0.001, Student’s *t*-test).
(TIF)

S1 Table The primers used for the construction of GETV mutants. (DOCX)
