# Supplementary material for: Defining the impact of Getah virus envelope protein glycosylation site mutations on viral replication, host adaptation, virulence, and immune evasion
Source: PLoS Pathog. 2026 Apr 2;22(4):e1014126. doi: 10.1371/journal.ppat.1014126 (PMC13068331; doi:10.1371/journal.ppat.1014126)
Supplement: S1 Table — (DOCX) [file ppat.1014126.s006.docx]

S1 Table The primers used for the construction of GETV mutants.

| Primer name | Primer sequence (5′−3′) | Purpose |
| --- | --- | --- |
| SrfI-F | CAACGTGGACCGCCCGGGCTACTA | Primers for the construction of the glycosylation site mutant virus at position 200 of the E2 protein |
| E2-200F | CAGATACcaaTGCACGTG |  |
| E2-200R | CACGTGCAttgGTATCTG |  |
| BstbI-R | RGGTGGCTACATTCGAATGAGAATGG |  |
| SrfI-F | CAACGTGGACCGCCCGGGCTACTA | Primers for the construction of the glycosylation site mutant virus at position 262 of the E2 protein |
| E2-262 F | CTCTGACCcaaTCCACAT |  |
| E2-262 R | ATGTGGAttgGGTCAGAG |  |
| BstbI-R | RGGTGGCTACATTCGAATGAGAATGG |  |
| SrfI-F | CAACGTGGACCGCCCGGGCTACTA | Primers for the construction of the glycosylation site mutant virus at position 141 of the E1 protein |
| E1-141 F | CGGGAACCTCcaaCAGAC |  |
| E1-141 R | GTCTGttgGAGGTTCCCG |  |
| BstbI-R | GGTGGCTACATTCGAATGAGAATGG |  |
| qE2-F | AGCGACAAGACTATCAATTCGT | Primers for the quantification of viral gene copy numbers |
| qE2-R | TGCACTTTACCTTTGCGAGAC |  |
